# Buyang Huanwu Decoction Enhances Revascularization via Akt/GSK3*β*/NRF2 Pathway in Diabetic Hindlimb Ischemia

**DOI:** 10.1155/2021/1470829

**Published:** 2021-12-03

**Authors:** Xiao-Yi Bao, Li-Hui Deng, Zi-Jun Huang, Abdirizak S. Daror, Zi-Hao Wang, Wang-Jun Jin, Zhuang Zhuang, Qiang Tong, Guo-Qing Zheng, Yan Wang

**Affiliations:** ^1^Department of Cardiology, The Second Affiliated Hospital and Yuying Children's Hospital of Wenzhou Medical University, Wenzhou 325027, China; ^2^Department of Cardiology, The First Affiliated Hospital of Zhejiang Chinese Medical University (Zhejiang Provincial Hospital of Chinese Medicine), China

## Abstract

**Background:**

Peripheral arterial disease (PAD) is a typical disease of atherosclerosis, most commonly influencing the lower extremities. In patients with PAD, revascularization remains a preferred treatment strategy. Buyang Huanwu decoction (BHD) is a popular Chinese herbal prescription which has showed effects of cardiovascular protection through conducting antioxidant, antiapoptotic, and anti-inflammatory effects. Here, we intend to study the effect of BHD on promoting revascularization via the Akt/GSK3*β*/NRF2 pathway in diabetic hindlimb ischemia (HLI) model of mice.

**Materials and Methods:**

All db/db mice (*n* = 60) were randomly divided into 6 groups by table of random number. (1) Sham group (*N* = 10): 7-0 suture thread passed through the underneath of the femoral artery and vein without occlusion. The remaining 5 groups were treated differently on the basis of the HLI (the femoral artery and vein from the inguinal ligament to the knee joint were transected and the vascular stump was ligated with 7-0 silk sutures) model: (2) HLI+NS group (*N* = 15): 0.2 ml NS was gavaged daily for 3 days before modeling and 14 days after occlusion; (3) HLI+BHD group (*N* = 15): 0.2 ml BHD (20 g/kg/day) was gavaged daily for 3 days before modeling and 14 days after occlusion; (4) HLI+BHD+sh-NC group (*N* = 8): local injection of adenovirus vector carrying the nonsense shRNA (Ad-GFP) in the hindlimbs of mice before treatment; (5) HLI+BHD+sh-NRF2 group (*N* = 8): knockdown of NRF2 in the hindlimbs of mice by local intramuscular injection of adenovirus vector carrying NRF2 shRNA (Ad-NRF2-shRNA) before treatment; and (6) HLI+BHD+LY294002 group (*N* = 4): intravenous injection of LY294002 (1.5 mg/kg) once a day for 14 days on the basis of the HLI+BHD group. Laser Doppler examination, vascular cast, and immunofluorescence staining were applied to detect the revascularization of lower limbs in mice. Western blot analysis was used to detect the expression of vascular endothelial growth factor (VEGF), interleukin-1beta (IL-1*β*), interleukin-6 (IL-6), tumor necrosis factor- (TNF-) *α*, heme oxygenase-1 (HO-1), NAD(P)H dehydrogenase quinone-1 (NQO-1), catalase (CAT), glyceraldehyde-3-phosphate dehydrogenase (GAPDH), phosphorylated protein kinase B (p-AKT), and phosphorylated glycogen synthase kinase-3 beta (p-GSK3*β*). HE staining was used to assess the level of muscle tissue damage and inflammation in the lower extremities. Local multipoint injection of Ad-NRF2-shRNA was used to knock down NRF2, and qPCR was applied to detect the mRNA level of NRF2. The blood glucose, triglyceride, cholesterol, MDA, and SOD levels of mice were tested using corresponding kits. The SPSS 20.0 software and GraphPad Prism 6.05 were used to do all statistics. Values of *P* < 0.05 were considered as statistically significant. *Results and Conclusions*. BHD could enhance the revascularization of lower limbs in HLI mice, while BHD has no effect on blood glucose and lipid level in db/db mice (*P* > 0.05). BHD could elevate the protein expression of VEGF, HO-1, NQO-1, and CAT (*P* < 0.05) and decrease the expression of IL-1*β*, IL-6, and TNF-*α* (*P* < 0.05) in HLI mice. Meanwhile, BHD could activate NRF2 and promote the phosphorylation of AKT/GSK3*β* during revascularization (*P* < 0.05). In contrast, knockdown of NRF2 impaired the protective effects of BHD on HLI (*P* < 0.05). LY294002 inhibited the upregulation of NRF2 activated by BHD through inhibiting the phosphorylation of the AKT/GSK3*β* pathway (*P* < 0.05). The present study demonstrated that BHD could promote revascularization on db/db mice with HLI through targeting antioxidation, anti-inflammation, and angiogenesis via the AKT/GSK3*β*/NRF2 pathway.

## 1. Introduction

Diabetes mellitus (DM) is a group of metabolic diseases characterized by high glucose and insufficient insulin secretion and/or dysfunction, which leads to chronic damage, dysfunction, and even failure of multiple organs. Surveys from the World Health Organization and International Diabetes Federation demonstrated that the global prevalence of DM has reached 8.5%, which affects about 450 million people [1]. The prevalence of DM imposes a heavy social and economic burden on the world. In 2017, approximately 4 million patients worldwide died of DM, and the related expenditures were close to $727 billion [2]. In China, this situation is even more serious. More than 840,000 people died of DM in 2017, accounting for 21% of global diabetes deaths [1]. The leading causes of in-hospital and death of DM patients are microvascular and macrovascular complications, accounting for 80% of diabetes-related deaths [3]. Peripheral artery disease (PAD), a common vessel complication of DM, is featured as impaired blood flow and ischemia caused by partial or complete occlusion of one or more noncoronary arteries, most often involving the lower extremities [4]. As PAD progresses, intermittent claudication (IC) usually occurs in the early stages, and in its most advanced stage, PAD can lead to critical limb ischemia (CLI), amputation, and even death [5]. Intervention of risk factors, optimal drug therapy, and individualized exercise are the first-line treatments in IC. For patients with severe symptoms or CLI, surgery and catheter-based revascularization are the preferred treatments [6]. These procedures ensure that approximately 75% of patients have more than one year of survival and limb retention [7]. However, there are still one-third of PAD patients that are not suitable for bypass surgery or percutaneous transluminal angioplasty (PTA) due to excessive surgery risk or disadvantageous vascular condition [8]. Thus, it is still necessary to develop a new nonoperative strategy to promote the revascularization of ischemic limbs [9].

Angiogenesis (i.e., new capillaries growing from preexisting vessels and then forming capillary networks to expand blood flow distribution in ischemic tissues downstream of the arterial occlusion) [10] is an endogenous process, which can partially compensate for the insufficient tissue perfusion. For approximately 2 decades, “therapeutic angiogenesis” has been studied as an investigational approach to treat patients with symptomatic PAD [11]. Although several angiogenesis therapies are effective in animal models, the transformations in the clinic are not ideal [12–14]. Previous studies indicated that the ability of endogenous functional angiogenesis and the effectiveness of angiogenesis therapy significantly reduced in PAD patients [9, 15, 16]. This situation is closely associated with the complex pathophysiological changes of hyperglycemia, oxidative stress, and chronic inflammation [17]. Thus, there is an urgent need to restore blood flow to ischemic tissues while avoiding detrimental inflammation and other side effects.

Increasingly, evidence has demonstrated that Chinese Herbal Medicines (CHM) have a protection effect on ischemic disease via targeting angiogenesis [18–20]. Buyang Huanwu decoction (BHD), originally recorded in Yilin Gaicuo, is a classic Chinese herbal prescription, which has been utilized for treating vascular diseases for hundreds of years. BHD is made up of seven Chinese herbs: (1) Huang Qi, (2) Dang Gui, (3) Chi Shao, (4) Chuan Xiong, (5) Hong Hua, (6) Tao Ren, and (7) Di Long, and the detail information about these drugs was recorded in our previous study [21]. Based on traditional Chinese medicine theory, BHD has the function of invigorating the body, enhancing blood circulation, and activating Qi flow through energy meridians. Accumulating evidences demonstrated that BHD could improve the outcomes of ischemic disease [22–24]. Modern pharmacological studies suggested that the protective effects of BHD in ischemic diseases may involve the following mechanisms: anti-inflammation [25, 26], antithrombus [27, 28], and antioxidation [29–31]. Other studies also showed that BHD could promote angiogenesis through increasing the expression of VEGF, VEGFR2, Flk-1, bFGF, and angiopoietin-1 (Ang-1) in ischemic cardiocerebral disease models [21, 23, 32–37]. The fact that different pathological phenotypes have the same molecular mechanism provides a novel theory of therapy concept that is concluded as “several diseases, one drug” and promotes drug repurposing [38]. Accordingly, we speculate that BHD could improve the complex microenvironmental state of PAD patients while targeting angiogenesis to promote revascularization.

Nuclear factor- (erythroid-derived 2-) like 2 (NRF2), belonging to the cap'n'collar family, is a kind of leucine zipper transcription factor and is also one primary molecule for protecting and recovering the cellular environmental homeostasis [17]. NRF2 could regulate the transcription of multiple antioxidant genes, such as heme oxygenase-1 (HO-1) and NAD(P)H dehydrogenase quinone 1 (NQO-1) [17, 39]. In addition, a previous study indicated that the activation of NRF2 could confront inflammation [40]. NRF2 not only could directly bind to the promoter of inflammatory factors to inhibit their transcription [41] but also regulates the expression of NF-KB in a crosstalk manner [42]. Recent studies indicated that NRF2 played a key role in angiogenesis, and the lack of NRF2 would lower the survival and proliferation of endothelial cells and the function of angiogenesis [43]. In NRF2-knockout rats, the ability of cardiac microvascular production was also impaired [44]. In the early stage of DM, the expression of NRF2 was increased, which is a natural defense mechanism in an injured organism for confronting high glucose. Accumulating evidences indicated that the activation of NRF2 in diabetes or its complications was beneficial [17]. Therefore, from a clinical point of view, it is of great significance to activate NRF2 by pharmacological methods for patients with PAD [45]. GSK3*β*-mediated phosphorylation is one of the main ways to regulate the activity of NRF2, which strictly controls the expression of NRF2 at the level of cell signal transduction. Recent studies have confirmed that the phosphorylation of AKT could inhibit the activity of GSK3*β* and promote the activation and nuclear translocation of NRF2 [3, 16, 33, 46, 47].

Extrapolating from those studies, we hypothesized that BHD could promote the revascularization of PAD through AKT/GSK3*β*-mediated NRF2 activation.

## 2. Materials and Methods

### 2.1. Animals

A total of 60 db/db male mice, aged at 6-8 weeks, with C57BL/6 background, were purchased from Jiangsu Jicui Pharmaceutical Health Biotechnology Co., Ltd., and housed in the laboratory animal center of Wenzhou Medical University. The animals were maintained on standard chow and tap water, which were available ad libitum, and were in a temperature-controlled chamber at 24°C with a 12 h light–dark cycle. All animal experimental procedure protocols were approved by the Animal Ethics Committee of the laboratory animal center of Wenzhou Medical University (number wydw2014-0058).

### 2.2. Drugs and Reagents

BHD consists of seven herbal medicines which include Huang Qi, Dang Gui, Chi Shao, Chuan Xiong, Tao Ren, Hong Hua, and Di Long, with the ratio of 120 : 6 : 5 : 3 : 3 : 3 : 3, and the specific formulation was described previously [20]; CD31 polyclonal antibody (ab28364) and VEGF polyclonal antibody (ab46154) were purchased from Abcam (UK); NAD(P)H dehydrogenase quinone 1 (NQO-1) polyclonal antibody (DF6437) was purchased from Affinity Biosciences (USA); heme oxygenase-1 (HO-1) polyclonal antibody (10701-1-AP), catalase polyclonal antibody (21260-1-AP), and histone H3 (17168-1-AP) were purchased from Proteintech (CHN); NRF2 monoclonal antibody (12721), Akt (4691) and phospho-Akt monoclonal antibody (4060), glycogen synthase kinase-3 beta (GSK3*β*) (12456) and phospho-GSK3*β* (5558) monoclonal antibody, interleukin-6 (IL-6) monoclonal antibody (12912), interleukin-1beta (IL-1*β*) monoclonal antibody (12242), tumor necrosis factor- (TNF-) *α* monoclonal antibody (11948), and glyceraldehyde-3-phosphate dehydrogenase (GAPDH) monoclonal antibody (5174) were purchased from Cell Signaling Technology (USA).

### 2.3. Hindlimb Ischemia (HLI) Model Establishment

The HLI model was established with reference to the previous publication [9]. Briefly, mice were anesthetized with isoflurane (2% isoflurane in 100% oxygen at a floodwater of 1 l/min) and maintained on a hot plate with temperature controlled at 37°C. After fixation, the hindlimbs were shaved and the operation area was sterilized with iodophor. A longitudinal incision was made in the left groin to completely expose the femoral artery and vein. Then, the vessels from the inguinal ligament to the knee joint were transected, and the vascular stump was ligated with 7-0 silk sutures. The skin incision was closed using 5-0 silk sutures. All db/db mice (*n* = 60) were randomly divided into 6 groups by table of random number. (1) Sham group (*N* = 10): 7-0 suture thread passed through underneath of the femoral artery and vein without occlusion. The remaining 5 groups were treated differently on the basis of the HLI (the femoral artery and vein from the inguinal ligament to the knee joint were transected and the vascular stump were ligated with 7-0 silk sutures) model: (2) HLI+NS group (*N* = 15): 0.2 ml NS was gavaged daily for 3 days before modeling and 14 days after occlusion; (3) HLI+BHD group (*N* = 15): 0.2 ml BHD (20 g/kg/day) was gavaged daily for 3 days before modeling and 14 days after occlusion; (4) HLI+BHD+sh-NC group (*N* = 8): local injection of adenovirus vector carrying the nonsense shRNA (Ad-GFP) in the hindlimbs of mice before treatment; (5) HLI+BHD+sh-NRF2 group (*N* = 8): knockdown of NRF2 in the hindlimbs of mice by local intramuscular injection of adenovirus vector carrying NRF2 shRNA (Ad-NRF2-shRNA) before treatment; and (6) HLI+BHD+LY294002 group (*N* = 4): intravenous injection of LY294002 (1.5 mg/kg) once a day for 14 days on the basis of the HLI+BHD group. Laser Doppler examination, vascular cast, and immunofluorescence staining were applied to detect the revascularization of lower limbs in mice.

### 2.4. Laser Doppler Examination

On the 0th, 7th, and 14th day after modeling, the mice were anesthetized with 2% isoflurane and fixed in the prone position. Laser Doppler examination was performed on each experimental group in sequence (Doppler blood flow monitoring system, Perimed, USA). The average perfusion (AP) value of the left and right lower extremities was measured, and the corresponding perfusion value percentage was calculated. All measurements were done by an experienced ultrasound technician who was blinded to the experimental design.

### 2.5. Determination of the Level of Blood Glucose

After fasting for 12 h, the blood glucose of db/db mice was measured at 9:00 on the 0th, 7th, and 14th day after modeling. In addition, after intragastric administration of NS or BHD to the mice, blood glucose was also measured at 60 min, 120 min, and 180 min. All the blood samples were collected from the tail of the mice and measured with Accu-Chek Active Test Strips (Roche Diagnostics GmbH, Germany).

### 2.6. Serum Biochemistry Analysis

On the 14th day after modeling, the levels of superoxide dismutase (SOD), malondialdehyde (MDA), triglyceride (TG), and total cholesterol (T-CHO) in serum were measured using commercial kits (Nanjing Jiancheng Bioengineering Institute, Nanjing, China). All the blood samples were collected from the portal vein of the mice and centrifuged at 3000 rpm for 15 min. Serum was stored in aliquots at -80°C for subsequent biochemical analysis.

### 2.7. Knockdown of NRF2

To knock down NRF2 expression in the hindlimbs of db/db mice, the adenovirus vector containing shRNA against NRF2 (Ad-NRF2-shRNA) was injected at two sites of the adductor and gastrocnemius muscle with 2 × 10^8^ plaque forming units [48]. As a control group, other adenovirus vectors containing nonsense shRNA without NRF2 knockdown function (Ad-GFP) were injected in the same manner and dose. The adenovirus vector containing shRNA against NRF2 or nonsense shRNA was constructed by Celluloid Biotechnology Co., Ltd. (Hangzhou, China). The specific target sequences are as follows: NRF2-shRNA: 5′-CTTGAAGTCTTCAGCATGTTA-3′; nonsense shRNA: 5′-CCTAAGGTTAAGTCGCCCTCG-3′.

The transfection was performed following the procedure described above. 48 h after transfection, the expression of NRF2 was determined by qPCR.

### 2.8. Muscle Histopathology

The muscle of ischemic area in the left hindlimb was collected and fixed in 4% paraformaldehyde-buffered solution and then embedded in paraffin according to standard procedure. Sections with 5 *μ*m thick slices were cut and then stained with hematoxylin and eosin (HE) solution. The slides were then viewed under an optical microscope (Olympus, Japan), and micrographs were taken.

### 2.9. Vascular Cast of Hindlimb

On the 14th day after modeling, the mice were anesthetized with isoflurane and fixed in a supine position on a modeling table. Shaving the chest area, the heart was fully exposed by tissue scissors. After removing the right atrial appendage, the left ventricle was injected with 20 ml of sodium heparin and sodium nitroprusside (1 : 1) mixed solution and 5 ml of 4% paraformaldehyde in order. Then, 2.4 ml of contrast agent (MICROFIL, Flow Tech, USA) was slowly injected. After the contrast agent was solidified, the lower limbs to be tested were taken. The specimen was put in 4% paraformaldehyde for 48 h and then decalcified with EDTA. Finally, micro-CT (*μ*CT100, SCANCO, Switzerland) was used to perform three-dimensional reconstruction of lower extremity blood vessels, and images of revascularization of lower limbs were obtained.

### 2.10. Western Blot Analysis

Total protein isolated from the muscle of ischemic area was uniformly mixed with SDS-PAGE sample loading buffer, boiled for 10 min, and transferred to a polyvinylidene difluoride (PVDF) membrane. The membranes were then blocked with 5% nonfat milk diluted by TBST for 2 h at room temperature and then incubated with primary antibodies including VEGF (1 : 1000), IL-1*β* (1 : 1000), IL-6 (1 : 1000), TNF-*α* (1 : 1000), HO-1 (1 : 1000), NQO-1 (1 : 1000), CAT (1 : 1000), GAPDH (1 : 5000), p-AKT(1 : 1000), and p-GSK3*β* (1 : 1000) at 4°C overnight. The next day, membranes were washed three times with TBST for 10 min and then further incubated with secondary antibodies (1 : 10000) for 2 h at room temperature. The membranes were washed again, and blots were visualized by ChemiDoc™ XRS+ Imaging System. The relative protein levels were quantified by ImageJ software (NIH, Bethesda, MD, USA).

### 2.11. Immunofluorescence Staining

To measure the level of neovascularization in the ischemic area, the samples were sectioned in 5 *μ*m slices and the antigens in tissue sections were repaired by sodium citrate buffer at 100°C. After blocking the antigen with goat serum for 2 h, primary antibody CD-31 (1 : 100) and secondary antibody Alexa Flour 647 conjugated antibody (1 : 800) were used to incubate the tissues. Evaluation of the immunohistochemical staining was performed using a fluorescence microscope (Leica, Germany).

### 2.12. Real-Time Quantitative Reverse Transcription Polymerase Chain Reaction (RT-qPCR)

Total RNA was isolated with a TRIzol reagent according to the manufacturer's instructions (Invitrogen, USA). cDNA is synthesized using the PrimeScript™ RT Reagent Kit (TAKARA, Japan). Real-time polymerase chain reactions were performed with SYBR Green (Roche, Switzerland). The mRNA expression of NRF2 was performed using the LightCycler thermal cycler system (Bio-Rad, U.S.A) for quantitative RT-PCR. All data was normalized to the mRNA expression level of GAPDH. Gene-specific primers were as follows: NRF2: forward, 5′-TAGATGACCATGAGTCGCTTGC-3′, and reverse, 5′-GCCAAACTTGCTCCATGTCC-3′; GAPDH, the forward primer 5′-TCAACAGCAACTCCCACTCTTCCA-3′ and reverse primer 5′-ACCCTGTTGCTGTAGCCGTATTCA-3′.

### 2.13. Statistical Analysis

All data were expressed as mean ± standard deviation (SD). Comparisons of differences in continuous variables were conducted with Student's *t*-test, Mann–Whitney *U* test, one-way ANOVA test, or two-way ANOVA test with Bonferroni correction for multiple comparisons where appropriate. Statistical analysis was performed using SPSS 21.0 software and GraphPad Prism 6.05. A two-tailed value of *P* of 0.05 or less was considered to be statistically significant.

## 3. Results

### 3.1. BHD Has No Effect on Blood Glucose and Lipid in db/db Mice

**Figure 1:**
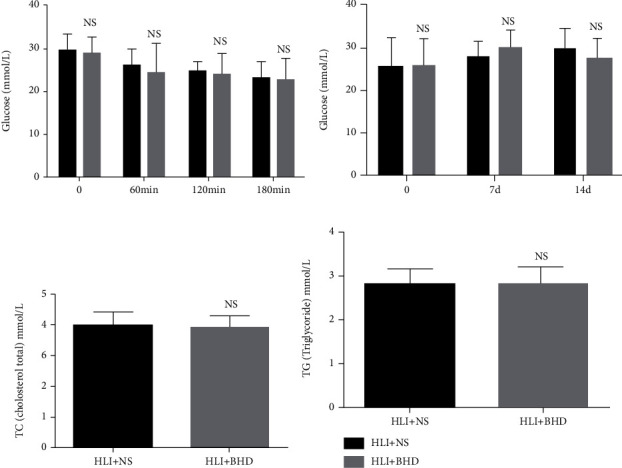
BHD has no effect on blood glucose and lipid in HLI mice. (a) The level of short-term blood glucose (*n* = 6). (b) The level of long-term blood glucose after 7 days and 14 days of BHD treatment (*n* = 6). Results in (a) and (b) were analyzed by repeated-measure two-way ANOVA. *Post hoc* Bonferroni test: NS, *P* > 0.05 compared with HLI+NS group. (c) The level of TC after 14 days of BHD treatment (*n* = 6). (d) The level of TG after 14 days of BHD treatment (*n* = 6). Mean ± SD. NS, *P* > 0.05, compared with HLI+NS group.

To clarify if BHD would influence the blood glucose level in db/db mice, short-term and long-term changes in blood glucose after administration were detected. The two-way ANOVA reveals that there was no significant difference in blood glucose between the HLI+NS group and the HLI+BHD group at 60 min, 120 min, and 180 min after administration (Figure 1(a); *P* > 0.05), and there was no interaction between drug administration and the time point of measurement for blood glucose (*F* (3, 36) = 0.3169, *P* = 0.8130). After 7 days and 14 days of continuous administration, two-way ANOVA shows that there was no significant difference in fasting blood glucose between the groups (fasting blood glucose was measured at 9 am) (Figure 1(b); *P* > 0.05), and there was no interaction between drug administration and the time point of measurement for blood glucose (*F* (2, 22) = 1.235, *P* = 0.3102). The levels of serum triglyceride and cholesterol after 14 days of administration were also detected. Compared with the HLI+NS group, no significant changes were observed in the HLI+BHD group (Figures 1(c) and 1(d); *P* > 0.05).

### 3.2. BHD Enhances Tissue Perfusion and Angiogenesis in HLI

**Figure 2:**
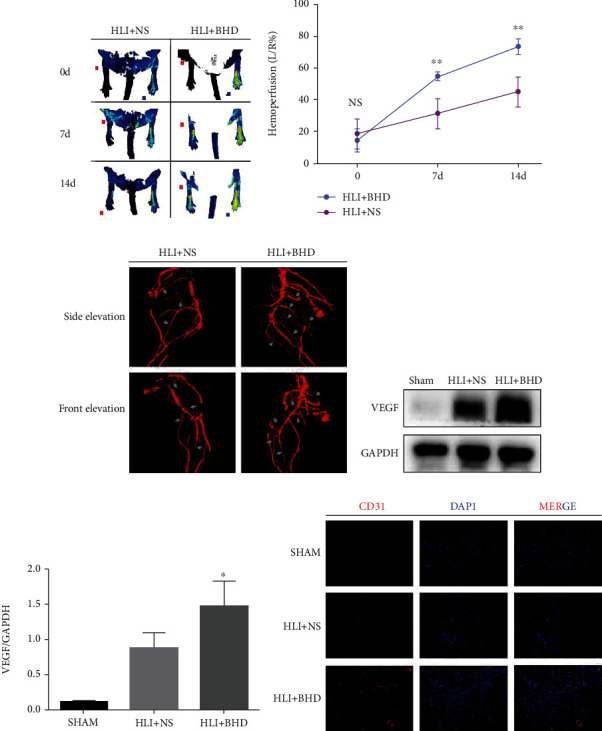
BHD increases tissue perfusion and angiogenesis. (a, b) The tissue perfusion level of the hindlimbs in the HLI mice after 0, 7, and 14 days of BHD treatment (*n* = 6). (c) Micro-CT imaging of tissue vessels in hindlimbs of HLI mice (*n* = 3). (d, e) The protein expression level and quantitative analysis of VEGFA after 14 days of BHD treatment (*n* = 4). (f) Angiogenesis in the ischemic area of the hindlimbs in HLI mice after 14 days of BHD treatment (*n* = 3). Mean ± SD. NS, *P* > 0.05, compared with HLI+NS group; ^∗^*P* < 0.05 and ^∗∗^*P* < 0.01, compared with HLI+NS group.

Laser Doppler perfusion imaging showed a time-dependent recovery and increase in tissue perfusion level in HLI mice, reaching 30% on day 14 after modeling (Figures 2(a) and 2(b)). However, mice treated with BHD showed significantly better improvement in perfusion on day 14 (Figures 2(a) and 2(b); *P* < 0.05). Vascular cast and micro-CT were used to visualize the limb vasculature. Reconstructed images of the vascular cast of limbs showed that there were more collateral vessels in the HLI+BHD group than the HLI+NS group, and the blood perfusion area in the HLI+BHD group was significantly increased on day 14 (Figure 2(c)). In further experiments, the expression of VEGFA in the ischemic region of the lower extremities was detected. The results of WB showed that the protein expression of VEGFA in the HLI+BHD group was significantly increased at 14 days (Figures 2(d) and 2(e); *P* < 0.05). Meanwhile, the immunofluorescence results of CD31-positive staining showed that the number of CD31-positive sites in the HLI+BHD group was significantly higher than that in the HLI+NS group, and there were more microvascular formations in the HLI+BHD group on day 14. (Figure 2(f)).

## 4. BHD Reduces Tissue Inflammation during Revascularization

**Figure 3:**
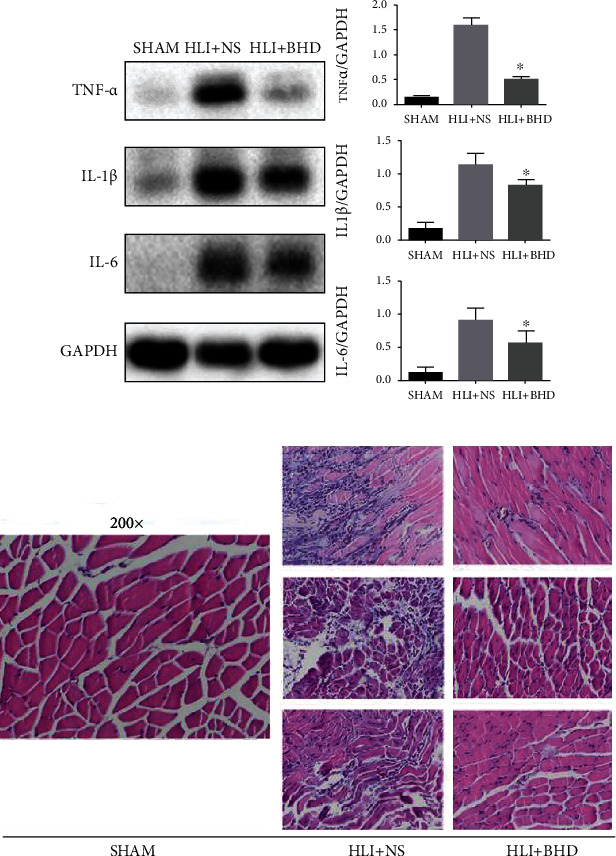
BHD reduces inflammation and improves tissue damage during revascularization. (a) The protein expression level and quantitative analysis of inflammatory factors in ischemic area of the hindlimbs in HLI mice after 14 days of BHD treatment (*n* = 4). (b) HE staining of the hindlimbs in HLI mice after 14 days of BHD treatment (*n* = 3). Mean ± SD. ^∗^*P* < 0.05, compared with HLI+NS group.

To test the effects of BHD on inflammation in the ischemic area of the hindlimb, the protein expressions of inflammatory factors IL-1*β*, IL-6, and TNF-*α* were detected by WB and the pathological changes of ischemic tissue were observed by HE staining on day 14. The results of WB showed that the protein expressions of IL-1*β*, IL-6, and TNF-*α* in the ischemic region of the hindlimbs were significantly decreased in the HLI+BHD group (Figure 3(a); *P* < 0.05). Furthermore, HE staining showed that, compared with the HLI+NS group, the ischemic tissue of the HLI+BHD group showed less inflammatory cell infiltration and more moderate damage (Figure 3(b)).

## 5. BHD Attenuates Oxidative Stress during Revascularization

**Figure 4:**
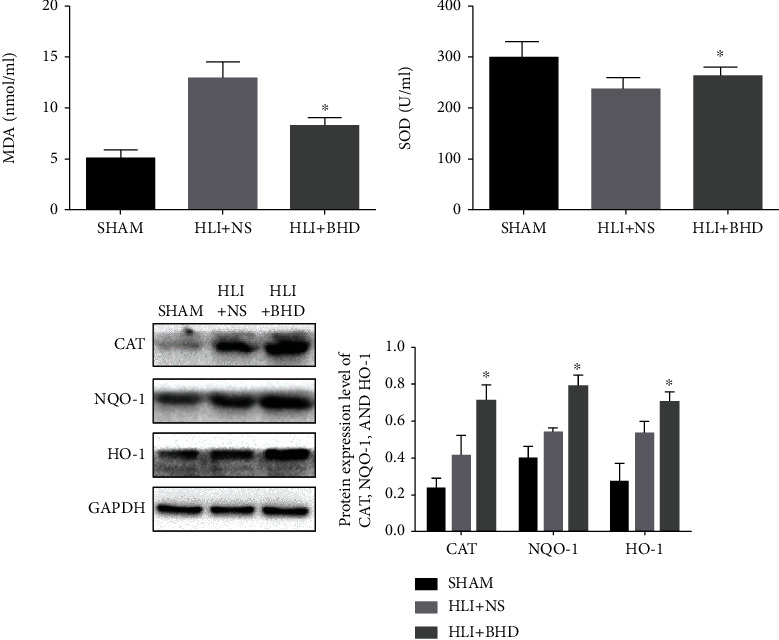
BHD reduces oxidative stress levels of ischemic area during revascularization. (a) The level of MDA in serum after 14 days of BHD treatment (*n* = 6). (b) The level of SOD in serum after 14 days of BHD treatment (*n* = 6). (c) The protein expression level of CAT, HO-1, and NQO-1 after 14 days of BHD treatment (*n* = 4). Mean ± SD. ^∗^*P* < 0.05 and ^∗∗^*P* < 0.01, compared with HLI+NS group.

To test the effects of BHD on the oxidative stress level in the HLI mice, the levels of SOD and MDA in the serum and the protein expression level of HO-1, NQO-1, and CAT in the ischemic area were detected on day 14. Serological tests showed a significant increase in the SOD level and a significant decrease in the MDA level in the HLI+BHD group (Figures 4(a) and 4(b); *P* < 0.05). The results of WB showed that the protein expression of HO-1, NQO-1, and CAT in the HLI+BHD group were significantly increased (Figure 4(c); *P* < 0.05).

## 6. BHD Could Activate NRF2 and Promote the Phosphorylation of AKT/GSK3*β* during Revascularization

**Figure 5:**
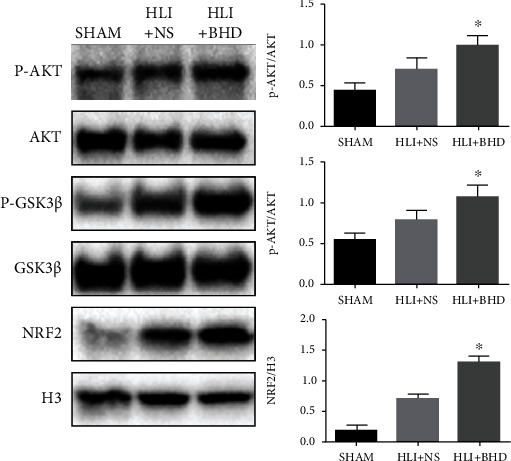
BHD activates NRF2 and promotes the phosphorylation of AKT/GSK3*β* during revascularization. The protein expression and quantitative analysis of NRF2 in nuclear and the phosphorylation and quantitative analysis of Akt and GSK3*β* of tissue in ischemic area after 14 days of BHD treatment (*n* = 4). Mean ± SD. ^∗^*P* < 0.05, compared with HLI+NS group.

To clarify the underlying mechanism of the protective effect of BHD on db/db mice with HLI, further experiments were carried out on day 14. The results of WB showed that the protein expression of NRF2 in nuclear was significantly increased in the HLI+BHD group (Figure 5; *P* < 0.05) and the phosphorylation level of AKT/GSK3*β* was also increased (Figure 5; *P* < 0.05).

## 7. Knockdown of NRF2 Impairs the Protective Effects of BHD on HLI

**Figure 6:**
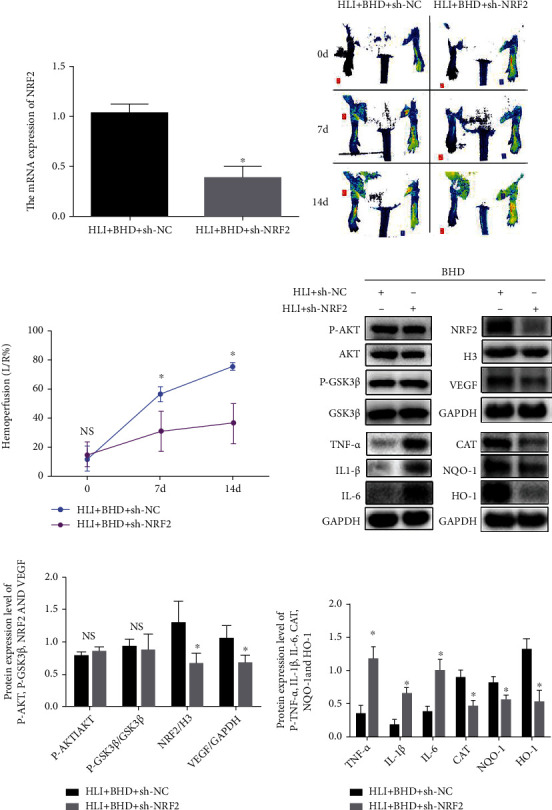
BHD promotes revascularization of hindlimb of HLI mice via AKT/GSK3*β*/NRF2 signal pathway. (a) The mRNA expression of NRF2 after injection of Ad-NRF2-shRNA (*n* = 4). (b, c) The tissue perfusion of hindlimb in HLI mice after 0, 7, and 14 days of BHD treatment (*n* = 4). (d–f) The protein expression and quantitative analysis of NRF2, VEGFA, TNF-*α*, IL-1*β*, IL-6, CAT, NQO-1, and HO-1 and the phosphorylation and quantitative analysis of Akt and GSK3*β* in ischemic area after 14 days of BHD treatment (*n* = 4). Mean ± SD. NS, *P* > 0.05; ^∗^*P* < 0.05, compared with HLI+BHD+sh-NRF2 group.

After confirming that Ad-NRF2-shRNA successfully knocked down NRF2 on the lower extremities of diabetic mice (Figure 6(a); *P* < 0.05), further experiments were carried out and corresponding indicators were tested at 14 days after modeling. Compared with the unknockdown group, there was a significant decrease in perfusion of the NRF2 knockdown group on the laser Doppler flowmeter (Figures 6(b) and 6(c); *P* < 0.05). WB showed that the phosphorylation level of AKT and GSK3*β* in the knockdown group has not significantly decreased (Figures 6(d) and 6(e); *P* > 0.05). However, the NRF2 activation and nuclear translocation were significantly attenuated (Figures 6(d) and 6(e); *P* < 0.05), and IL-1*β*, IL-6, and TNF-*α* impressions were significantly increased, compared with the unknockdown group (Figures 6(d) and 6(f); *P* < 0.05). Meanwhile, the expressions of HO-1, NQO-1, CAT, and VEGFA were significantly reduced (Figures 6(d) and 6(f); *P* < 0.05).

## 8. BHD Activates NRF2 via Akt/GSK3*β* Pathway

**Figure 7:**
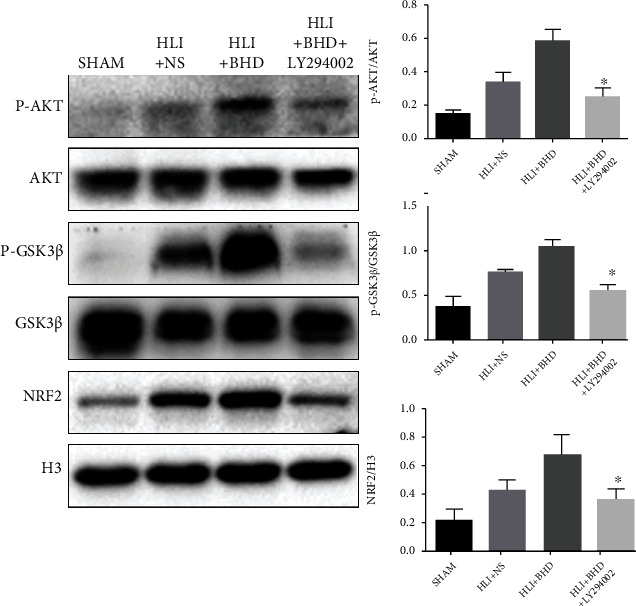
BHD activates NRF2 through Akt/GSK3*β* signal pathway. After injection of inhibitor, the protein expression and quantitative analysis of NRF2 in nuclear and the phosphorylation and quantitative analysis of Akt and GSK3*β* in ischemic area (*n* = 4). Mean ± SD. ^∗^*P* < 0.05, compared with HLI+BHD group.

NRF2 plays a key role in the response of cells to oxidative stress and could regulate the expression of inflammatory factors in an indirect or direct manner [3]. The results of WB showed that BHD significantly increased phosphorylation of Akt and GSK3*β* and ultimately promoted the activation and nuclear translocation of NRF2 on day 14 (Figure 7; *P* < 0.05). However, after the addition of LY294002, this effect of BHD was inhibited (Figure 7; *P* < 0.05). It suggested that the protective effect of BHD on diabetic HLI mice may be achieved by activating NRF2 via the Akt/GSK3*β* pathway.

## 9. Discussion

Owing to the various pathophysiological changes including hyperglycemia, hyperlipidemia, and insulin resistance as well as oxidative stress, inflammation, and endothelial dysfunction in lower extremities, diabetic patients with PAD are often difficult to get benefit from revascularization therapy [17]. Therefore, it is necessary to control these confounding factors while performing revascularization therapy. The present study provides novel evidences for the benefits of BHD in experimental PAD model (HLI) in type 2 diabetic mice. In the beginning, we found that BHD could enhance blood perfusion and angiogenesis in HLI db/db mice by increasing the expression of VEGF. In subsequent exploration, BHD was found to be able to improve the condition of oxidative stress and chronic inflammatory in diabetes. Finally, we confirmed that the above effects of BHD were achieved by activating NRF2 via the AKT/GSK3*β* pathway.

BHD consists of seven herbal medicines including Huang Qi, Dang Gui, Chi Shao, Chuan Xiong, Tao Ren, Hong Hua, and Di Long in the ratio of 120 : 6 : 5 : 3 : 3 : 3 : 3 and is mainly known for the treatment of blood stagnation caused by Qi deficiency [49]. These main compounds play different roles in the classic formula. Huang Qi, with the largest amount, is the sovereign herb for the purpose of treating blood stasis by invigorating Qi so as to activate blood circulation [50]. The main function of Dang Gui, Chi Shao, Chuan Xiong, Tao Ren, and Hong Hua in BHD is to remove blood stagnation, and Di Long is used for dredging meridians and activating collaterals [51]. In modern times, BHD is still widely used throughout China and elsewhere in the world for the treatment of ischemic diseases [52]. Modern pharmacological studies based on BHD have further confirmed that the protective effect of BHD in the treatment of ischemic diseases may be achieved through different molecular mechanisms such as anti-inflammatory and anti-antioxidative stress and promotion of angiogenesis [21]. Thus, we speculate that BHD also has a protective effect on ischemic diseases such as PAD that occur in the lower limb.

PAD is a common and severe clinical manifestation of atherosclerosis in the lower limb and is more likely to occur in patients with DM [53]. The functional limitations associated with PAD are related to hemodynamic changes [54]. Compromised peripheral blood supply in the lower extremities leads to tissue ischemia and hypoxia [16], combined with clinical phenotypes ranging from no symptom to IC or CLI (rest pain, arterial ulceration, or gangrene) [55]. The prognosis of CLI is quite poor, with 1-year mortality and major amputation rate of 30% and 25%, respectively [56]. According to current guidelines, surgical bypass techniques or endovascular approaches are the optimal treatment methods for CLI. These procedures ensure that approximately 75% of patients have more than one year of survival and limb retention [7]. However, there are still up to 50% of CLI patients who are not candidate for the abovementioned revascularization methods due to excessive surgery risk or disadvantageous vascular involvement [57]. Furthermore, for those who have undergone revascularization, the risks for cardiovascular and limb events persist for a long term [58]. These disappointing results have motivated a new clinical strategy called “therapeutic angiogenesis” which uses genic, molecular, and cellular-based approaches to induce endogenous revascularization [11]. Endogenous revascularization is a compensatory mechanism for the body to cope with insufficient tissue perfusion after large vessel occlusion. It consists of two main parts: (1) new capillaries grow from preexisting blood vessels to form a capillary network and (2) functional collateral arteries grow from preexisting arterioarteriolar anastomoses around the occlusion [10, 59]. For approximately two decades, growth factors such as VEGF, FGF, and HGF have been used to treat PAD in preclinical trials and have obtained some beneficial results. Nonetheless, the translation of therapeutic benefits found in animal models to substantial improvements in PAD patients has failed [60]. There are several factors that could be responsible for these disappointing results. First, PAD is caused by a complex pathological process, including but not limited to oxidative stress, inflammation, endothelial dysfunction, dyslipidemia, and hyperglycemia [61]. High levels of extracellular blood glucose and subsequent oxidative stress are upstream triggers for most of the tissue damage and serious long-term complications associated with PAD and will inhibit the ability of endothelial cells in angiogenesis [17, 62]. In addition, the chronic inflammation of diabetes impairs the endogenous revascularization and limits the effectiveness of revascularization therapy [9, 15]. Second, the animal models used in preclinical studies are usually not totally inconsistent with the characteristics of PAD in humans. They are more capable of forming collateral circulation after ischemia and are younger and healthier, with no PAD-related risk factors [63, 64]. Therefore, it is necessary to control these confounding factors while performing revascularization therapy. In the present study, we used type 2 diabetic mice to establish the model of HLI, which simulates multiple clinical features such as hyperglycemia, hyperlipidemia, insulin resistance, and endothelial dysfunction of PAD patients. Through the use of this model, we found that although BHD could not affect the level of blood glucose and blood lipid in db/db mice, it could promote the revascularization of hindlimbs by increasing the expression of VEGF and inhibiting excessive oxidative stress and chronic inflammation in the ischemic area.

Subsequently, we wanted to clarify the underlying mechanism of the protective effect of BHD on HLI db/db mice. In further exploration, we discovered enhanced nuclear translocation of NRF2 and increased phosphorylation levels of AKT/GSK3*β* in the BHD group. NRF2, owing a unique basic-leucine-zipper (bZIP) motif, belongs to a small family of transcription factors [65] and is central in mediating oxidative stress signal response originally [66]. In the stable state, NRF2 combines with Kelch sample related protein-1 (Keap1) in the cytoplasm. After being activated, NRF2 translocates to the nucleus and will recruit and activate a variety of antioxidant genes: heme oxygenase-1 (HO-1), NADPH quinone oxidoreductase (NQO-1), and catalase (CAT) [67]. In addition, NRF2 also plays an essential role in the regulation of inflammation. The interruption of the NRF2 pathway will increase the susceptibility to various inflammatory conditions [68]. The potential mechanism of the NRF2 gene on inflammation has been confirmed in multiple preclinical studies: (1) inhibiting the activation of NF-*κ*B by preventing the degradation of I*κ*B-*α*, increasing HO-1 expression and antioxidant defenses [40]; (2) inhibiting the activation of NLRP3 inflammasome, caspase-1 cleavage, and IL-1*β* generation in macrophages by increasing the expression of NQO1 [69]; (3) inducing an anti-inflammatory phenotype of CD8^+^ T cells, macrophages, and microglia through upregulation of the cysteine and GSH levels [38]; (4) inhibiting the migration/infiltration of immune cells by modulating the expression of VCAM1 and MMP9 [70, 71]; (5) directly binding to the promoter regions of proinflammatory cytokines such as IL-6 and IL-1*β* and inhibiting RNA Pol II recruitment [41]. Moreover, growing evidence demonstrated that NRF2 possesses a novel function called angiogenesis. Genetic ablation of NRF2 suppresses the proliferation and angiogenic function of endothelial cells in vitro and in vivo [43]. Furthermore, in the preclinical model of ischemic retinopathy, cerebral ischemia, and HLI, knockdown of NRF2 limits the effectiveness of angiogenesis therapy [72–74]. Importantly, the activation of NRF2 could promote the expression of angiogenic genes in wound biopsies in diabetic patients [75]. In summary, NRF2 nuclear translocation could induce multiple cytoprotective responses such as antioxidation, anti-inflammation, and proangiogenesis, which is consistent with the protective effect of BHD in HLI db/db mice. Therefore, we speculate that it might be a key pharmacological molecular target of BHD. In the present study, compared with the HLI group, BHD-treated hindlimb had a significantly higher level of NRF2, together with higher level of its downstream HO-1, NQO-1, CAT, and VEGF. Conversely, the expression of inflammatory factors including IL-1, IL-6, and TNF-*α* was significantly decreased. To further verify this point, the shRNA-mediated knockdown of NRF2 was performed in db/db mice. As the results showed, the antioxidation, anti-inflammation, and revascularization in response to BHD were completely abrogated. Therefore, the present study demonstrated that the protective effect of BHD on the HLI db/db mice was through the NRF2 pathway.

Finally, further investigation was conducted to clarify the potential regulation mechanism of BHD on NRF2. Several recent studies have showed that the pharmacological effect of BHD was related to the AKT pathway [32, 76, 77], which modulates the function of multiple downstream proteins involved in cellular proliferation, survival, metabolism, and angiogenesis [78]. In particular, AKT could be activated through the phosphorylation of its S473 residue, p-Akt (S473) consequently phosphorylating GSK3*β* at the S9 residue, and then suppressed the function GSK3*β* [79]. Furthermore, emerging evidence demonstrated that GSK3*β* was responsible for NRF2 degradation in a Keap1-independent manner, and the inhibition of its function could promote the activation of NRF2 [80–82] Thus, we speculate that BHD might phosphorylate the S9 residue of GSK*β* through the AKT pathway, then leading to the activation NRF2. In the present study, we demonstrated that the activation of NRF2 induced by BHD was dependent on the Akt/GSK3*β* pathway, as evidenced by the significantly decreased NRF2 protein level after treatment with the Akt/GSK3*β* inhibitor LY294002.

## 10. Conclusion

The present study demonstrated that BHD could promote revascularization on db/db mice with HLI through targeting of antioxidation, anti-inflammation, and angiogenesis via the AKT/GSK3*β*/NRF2 pathway.

## Data Availability

The data used to support the findings of this study are available from the corresponding author upon request.

## References

[1] Huang Y., Karuranga S., Malanda B., Williams D. R. R. (2018). Call for data contribution to the IDF diabetes atlas 9th edition 2019. *Diabetes Research and Clinical Practice*.

[2] Cho N. H., Shaw J. E., Karuranga S. (2018). IDF diabetes atlas: global estimates of diabetes prevalence for 2017 and projections for 2045. *Diabetes Research and Clinical Practice*.

[3] Dai X., Yan X., Zeng J. (2017). Elevating CXCR7 improves angiogenic function of EPCs via Akt/GSK-3*β*/Fyn-Mediated Nrf2 activation in diabetic limb ischemia. *Circulation Research*.

[4] Morcos R., Louka B., Tseng A. (2018). The evolving treatment of peripheral arterial disease through guideline-directed recommendations. *Journal of Clinical Medicine*.

[5] Criqui M. H., Aboyans V. (2015). Epidemiology of peripheral artery disease. *Circulation Research*.

[6] Hiramoto J. S., Teraa M., de Borst G. J., Conte M. S. (2018). Interventions for lower extremity peripheral artery disease. *Nature Reviews. Cardiology*.

[7] Berceli S. A., Hevelone N. D., Lipsitz S. R. (2007). Surgical and endovascular revision of infrainguinal vein bypass grafts: analysis of midterm outcomes from the PREVENT III trial. *Journal of Vascular Surgery*.

[8] Rigato M., Monami M., Fadini G. P. (2017). Autologous cell therapy for peripheral arterial disease: systematic review and meta-analysis of randomized, nonrandomized, and noncontrolled studies. *Circulation Research*.

[9] Zhang M. J., Sansbury B. E., Hellmann J. (2016). Resolvin D2 enhances postischemic revascularization while resolving inflammation. *Circulation*.

[10] Semenza G. L. (2007). Vasculogenesis, angiogenesis, and arteriogenesis: mechanisms of blood vessel formation and remodeling. *Journal of Cellular Biochemistry*.

[11] Iyer S. R., Annex B. H. (2017). Therapeutic angiogenesis for peripheral artery disease: lessons learned in translational science. *JACC: Basic to Translational Science*.

[12] Al Sabti H. (2007). Therapeutic angiogenesis in cardiovascular disease. *Journal of Cardiothoracic Surgery*.

[13] Annex B. H. (2013). Therapeutic angiogenesis for critical limb ischaemia. *Nature Reviews. Cardiology*.

[14] Zachman A. L., Wang X., Tucker-Schwartz J. M. (2014). Uncoupling angiogenesis and inflammation in peripheral artery disease with therapeutic peptide-loaded microgels. *Biomaterials*.

[15] Cuadrado A., Martín-Moldes Z., Ye J., Lastres-Becker I. (2014). Transcription Factors NRF2 and NF-*κ*B Are Coordinated Effectors of the Rho Family, GTP-binding Protein RAC1 during Inflammation∗. *The Journal of Biological Chemistry*.

[16] López-Díez R., Shen X., Daffu G. (2017). AgerDeletion enhances ischemic muscle inflammation, angiogenesis, and blood flow recovery in diabetic mice. *Arteriosclerosis, Thrombosis, and Vascular Biology*.

[17] Matzinger M., Fischhuber K., Heiss E. H. (2018). Activation of Nrf2 signaling by natural products-can it alleviate diabetes?. *Biotechnology Advances*.

[18] Guo D., Murdoch C. E., Liu T. (2018). Therapeutic angiogenesis of Chinese herbal medicines in ischemic heart disease: a review. *Frontiers in Pharmacology*.

[19] Yu L. J., Zhang K. J., Zhu J. Z. (2017). Salvianolic acid exerts cardioprotection through promoting angiogenesis in animal models of acute myocardial infarction: preclinical evidence. *Oxidative Medicine and Cellular Longevity*.

[20] Zhang K. J., Zhu J. Z., Bao X. Y., Zheng Q., Zheng G. Q., Wang Y. (2017). Shexiang Baoxin pills for coronary heart disease in animal models: preclinical evidence and promoting angiogenesis mechanism. *Frontiers in Pharmacology*.

[21] Zhu J. Z., Bao X. Y., Zheng Q. (2019). Buyang Huanwu decoction exerts cardioprotective effects through targeting angiogenesis via caveolin-1/VEGF signaling pathway in mice with acute myocardial infarction. *Oxidative Medicine and Cellular Longevity*.

[22] Carozzi S., Nasini M. G., Schelotto C. (1990). Peritoneal dialysis fluid (PDF) C++ and 1,25(OH)2D3 modulate peritoneal macrophage (PM0) antimicrobial activity in CAPD patients. *Advances in Peritoneal Dialysis*.

[23] Cai G., Liu B., Liu W. (2007). Buyang Huanwu decoction can improve recovery of neurological function, reduce infarction volume, stimulate neural proliferation and modulate VEGF and Flk1 expressions in transient focal cerebral ischaemic rat brains. *Journal of Ethnopharmacology*.

[24] Ye R.-Q., Lin G.-B., Xie J.-J., Zeng J.-B., Song X.-R., Yang Y. (2009). Effects of Buyang Huanwu decoction on angiopoietin 1 and its receptor Tie-2 in patients with early diabetic nephropathy.

[25] Fan L. H., Wang K. Z., Cheng B. (2006). Effects of Buyang Huanwu decoction on apoptosis of nervous cells and expressions of Bcl-2 and bax in the spinal cord of ischemia reperfusion injury in rabbits. *Journal of Traditional Chinese Medicine*.

[26] Wang W. R., Lin R., Zhang H. (2011). The effects of Buyang Huanwu decoction on hemorheological disorders and energy metabolism in rats with coronary heart disease. *Journal of Ethnopharmacology*.

[27] Wu Y., Yang J. J., Ye C. J. (2015). Effect of Buyang Huanwu decoction on 32 cases of diabetic lower limb vascular disease. *Journal of New Chinese Medicine*.

[28] Zhang J. P., Li C. L., Guo X. X., Wang G. (2001). Effect of Buyang Huanwu decoction on platelet activating factor content in arterial blood pre- and post-arterial thrombosis in rats. *Journal of Traditional Chinese Medicine*.

[29] Cui H., Liu T., Li P. (2018). An intersectional study of lncRNAs and mRNAs reveals the potential therapeutic targets of Buyang Huanwu decoction in experimental intracerebral hemorrhage. *Cellular Physiology and Biochemistry*.

[30] Liu Y., Lin R., Shi X. (2011). The roles of Buyang Huanwu decoction in anti-inflammation, antioxidation and regulation of lipid metabolism in rats with myocardial ischemia. *Evidence-based Complementary and Alternative Medicine*.

[31] Shen J., Zhu Y., Huang K. (2016). Buyang Huanwu decoction attenuates H_2_O_2_-induced apoptosis by inhibiting reactive oxygen species-mediated mitochondrial dysfunction pathway in human umbilical vein endothelial cells. *BMC Complementary and Alternative Medicine*.

[32] Cui H. J., Yang A. L., Zhou H. J. (2015). Buyang Huanwu decoction promotes angiogenesis via vascular endothelial growth factor receptor-2 activation through the PI3K/Akt pathway in a mouse model of intracerebral hemorrhage. *BMC Complementary and Alternative Medicine*.

[33] Guo Q., Zhong M., Xu H., Mao X., Zhang Y., Lin N. (2015). A systems biology perspective on the molecular mechanisms underlying the therapeutic effects of Buyang Huanwu decoction on ischemic stroke. *Rejuvenation Research*.

[34] Liao F., Meng Y., Zheng H. (2018). Biospecific isolation and characterization of angiogenesis-promoting ingredients in Buyang Huanwu decoction using affinity chromatography on rat brain microvascular endothelial cells combined with solid-phase extraction, and HPLC-MS/MS. *Talanta*.

[35] Wei R. L., Teng H. J., Yin B. (2013). A systematic review and meta-analysis of Buyang Huanwu decoction in animal model of focal cerebral ischemia. *Evidence-based Complementary and Alternative Medicine*.

[36] Yang J., Gao F., Zhang Y., Liu Y., Zhang D. (2015). Buyang Huanwu decoction (BYHWD) enhances angiogenic effect of mesenchymal stem cell by upregulating VEGF expression after focal cerebral ischemia. *Journal of Molecular Neuroscience*.

[37] Zhang Z. Q., Tang T., Luo J. K. (2007). Effect of Qi-tonifying and stasis-eliminating therapy on expression of vascular endothelial growth factor and its receptors Flt-1, Flk-1 in the brain of intracerebral hemorrhagic rats. *Chinese Journal of Integrative Medicine*.

[38] Cuadrado A., Manda G., Hassan A. (2018). Transcription factor NRF2 as a therapeutic target for chronic diseases: a systems medicine approach. *Pharmacological Reviews*.

[39] Murakami S., Motohashi H. (2015). Roles of Nrf2 in cell proliferation and differentiation. *Free Radical Biology & Medicine*.

[40] Ahmed S. M., Luo L., Namani A., Wang X. J., Tang X. (2017). Nrf2 signaling pathway: pivotal roles in inflammation. *Biochimica et Biophysica Acta - Molecular Basis of Disease*.

[41] Kobayashi E. H., Suzuki T., Funayama R. (2016). Nrf2 suppresses macrophage inflammatory response by blocking proinflammatory cytokine transcription. *Nature Communications*.

[42] Wardyn J. D., Ponsford A. H., Sanderson C. M. (2015). Dissecting molecular cross-talk between Nrf2 and NF-kappaB response pathways. *Biochemical Society Transactions*.

[43] Florczyk U., Jazwa A., Maleszewska M. (2014). Nrf2 regulates angiogenesis: effect on endothelial cells, bone marrow-derived proangiogenic cells and hind limb ischemia. *Antioxidants & Redox Signaling*.

[44] Kuang L., Feng J., He G., Jing T. (2013). Knockdown of Nrf2 inhibits the angiogenesis of rat cardiac micro-vascular endothelial cells under hypoxic conditions. *International Journal of Biological Sciences*.

[45] Rada P., Rojo A. I., Evrard-Todeschi N. (2012). Structural and functional characterization of Nrf2 degradation by the glycogen synthase kinase 3/ -TrCP axis. *Molecular and Cellular Biology*.

[46] Buendia I., Michalska P., Navarro E., Gameiro I., Egea J., León R. (2016). Nrf2-ARE pathway: an emerging target against oxidative stress and neuroinflammation in neurodegenerative diseases. *Pharmacology & Therapeutics*.

[47] Lastres-Becker I., Innamorato N. G., Jaworski T. (2014). Fractalkine activates NRF2/NFE2L2 and heme oxygenase 1 to restrain tauopathy-induced microgliosis. *Brain*.

[48] Jiang L., Yin M., Wei X. (2015). Bach1 represses Wnt/*β*-Catenin signaling and angiogenesis. *Circulation Research*.

[49] Li J. H., Liu A. J., Li H. Q., Wang Y., Shang H. C., Zheng G. Q. (2014). Buyang Huanwu decoction for healthcare: evidence-based theoretical interpretations of treating different diseases with the same method and target of vascularity. *Evidence-based Complementary and Alternative Medicine*.

[50] Qi Y., Gao F., Hou L., Wan C. (2017). Anti-inflammatory and immunostimulatory activities of astragalosides. *The American Journal of Chinese Medicine*.

[51] Lee Y. S., Woo S. C., Kim S. Y., Park J. Y. (2020). Understanding the multi-herbal composition of Buyang Huanwu decoction: a review for better clinical use. *Journal of Ethnopharmacology*.

[52] Shen J., Huang K., Zhu Y., Xu K., Zhan R., Pan J. (2020). Buyang Huanwu decoction promotes angiogenesis after cerebral ischemia by inhibiting the Nox4/ROS pathway. *Evidence-based Complementary and Alternative Medicine*.

[53] Committee C. S. (1996). A randomised, blinded, trial of clopidogrel versus aspirin in patients at risk of ischaemic events (CAPRIE). *The Lancet*.

[54] Hiatt W. R., Armstrong E. J., Larson C. J., Brass E. P. (2015). Pathogenesis of the limb manifestations and exercise limitations in peripheral artery disease. *Circulation Research*.

[55] Walker T. G. (2009). Acute limb ischemia. *Techniques in Vascular and Interventional Radiology*.

[56] Benoit E., O'Donnell T. F., Iafrati M. D. (2011). The role of amputation as an outcome measure in cellular therapy for critical limb ischemia: implications for clinical trial design. *Journal of Translational Medicine*.

[57] Department of Cardiology Provincial Hospital affiliated to Shandong University, Shandong Province, China, Li M., Zhou H. (2013). Autologous bone marrow mononuclear cells transplant in patients with critical leg ischemia: preliminary clinical results. *Experimental and Clinical Transplantation*.

[58] Hess C. N., Wang T. Y., Weleski Fu J. (2020). Long-term outcomes and associations with major adverse limb events after peripheral artery revascularization. *Journal of the American College of Cardiology*.

[59] Grundmann S., Piek J. J., Pasterkamp G., Hoefer I. E. (2007). Arteriogenesis: basic mechanisms and therapeutic stimulation. *European Journal of Clinical Investigation*.

[60] Gorenoi V., Brehm M. U., Koch A., Hagen A., Cochrane Vascular Group (2017). Growth factors for angiogenesis in peripheral arterial disease. *Cochrane Database of Systematic Reviews*.

[61] Bartel D. P. (2004). MicroRNAs. *Cell*.

[62] Cooke J. P., Losordo D. W. (2015). Modulating the vascular response to limb ischemia: angiogenic and cell therapies. *Circulation Research*.

[63] Waters R. E., Terjung R. L., Peters K. G., Annex B. H. (2004). Preclinical models of human peripheral arterial occlusive disease: implications for investigation of therapeutic agents. *Journal of Applied Physiology*.

[64] Krishna S. M., Omer S. M., Golledge J. (2016). Evaluation of the clinical relevance and limitations of current pre-clinical models of peripheral artery disease. *Clinical Science (London, England)*.

[65] Shen Y., Liu X., Shi J., Wu X. (2019). Involvement of Nrf2 in myocardial ischemia and reperfusion injury. *International Journal of Biological Macromolecules*.

[66] Venugopal R., Jaiswal A. K. (1996). Nrf1 and Nrf2 positively and c-Fos and Fra1 negatively regulate the human antioxidant response element-mediated expression of NAD(P)H:quinone oxidoreductase1 gene. *Proceedings of the National Academy of Sciences*.

[67] Karan A., Bhakkiyalakshmi E., Jayasuriya R., Sarada D. V. L., Ramkumar K. M. (2020). The pivotal role of nuclear factor erythroid 2-related factor 2 in diabetes- induced endothelial dysfunction. *Pharmacological Research*.

[68] Jiang S., Yang Y., Li T. (2016). An overview of the mechanisms and novel roles of Nrf2 in cardiovascular diseases. *Expert Opinion on Therapeutic Targets*.

[69] Liu X., Zhang X., Ding Y. (2017). Nuclear factor E2-related factor-2 negatively regulates NLRP3 inflammasome activity by inhibiting reactive oxygen species-induced NLRP3 priming. *Antioxidants & Redox Signaling*.

[70] Bourdonnay E., Morzadec C., Fardel O., Vernhet L. (2009). Redox-sensitive regulation of gene expression in human primary macrophages exposed to inorganic arsenic. *Journal of Cellular Biochemistry*.

[71] Wenzel P., Rossmann H., Müller C. (2015). Heme oxygenase-1 suppresses a pro-inflammatory phenotype in monocytes and determines endothelial function and arterial hypertension in mice and humans. *European Heart Journal*.

[72] Chen Y., Zhang X., Yang Y. (2019). Tert-butylhydroquinone enhanced angiogenesis and astrocyte activation by activating nuclear factor-E2-related factor 2/heme oxygenase-1 after focal cerebral ischemia in mice. *Microvascular Research*.

[73] Hsieh M. H., Tsai H. W., Lin K. J. (2019). An in situ slow-releasing H_2_S donor depot with long-term therapeutic effects for treating ischemic diseases. *Materials Science & Engineering. C, Materials for Biological Applications*.

[74] Wei Y., Gong J., Xu Z. (2015). Nrf2 in ischemic neurons promotes retinal vascular regeneration through regulation of semaphorin 6A. *Proceedings of the National Academy of Sciences of the United States of America*.

[75] Dhamodharan U., Karan A., Sireesh D. (2019). Tissue-specific role of Nrf2 in the treatment of diabetic foot ulcers during hyperbaric oxygen therapy. *Free Radical Biology & Medicine*.

[76] Li Z., Wang H., Wang Q., Sun J. (2016). Buyang Huanwu decoction vigorously rescues PC12 cells against 6-OHDA-induced neurotoxicity via Akt/GSK3*β* pathway based on serum pharmacology methodology. *Rejuvenation Research*.

[77] Liu N., Jiang Y. Y., Huang T. T., Hou J. C., Liu J. X. (2018). A network pharmacology approach to explore mechanisms of Buyang Huanwu decoction for treatment of cerebral infarction. *Zhongguo Zhong Yao Za Zhi*.

[78] Rodon J., Dienstmann R., Serra V., Tabernero J. (2013). Development of PI3K inhibitors: lessons learned from early clinical trials. *Nature Reviews. Clinical Oncology*.

[79] Gong L., Zhang Q. L., Zhang N. (2012). Neuroprotection by urate on 6-OHDA-lesioned rat model of Parkinson's disease: linking to Akt/GSK3*β* signaling pathway. *Journal of Neurochemistry*.

[80] Li X., Zou Y., Xing J. (2020). Pretreatment with roxadustat (FG-4592) attenuates folic acid-induced kidney injury through antiferroptosis via Akt/GSK-3beta/Nrf2 pathway. *Oxidative Medicine and Cellular Longevity*.

[81] Sotolongo K., Ghiso J., Rostagno A. (2020). Nrf2 activation through the PI3K/GSK-3 axis protects neuronal cells from A*β*-mediated oxidative and metabolic damage. *Alzheimer's Research & Therapy*.

[82] Yang T., Sun Y., Mao L. (2018). Brain ischemic preconditioning protects against ischemic injury and preserves the blood-brain barrier _via_ oxidative signaling and Nrf2 activation. *Redox Biology*.

